# Evaluation of Pinopodes Expression on the Mouse Endometrium Immediately before Implantation by Treatment with HMG/HCG and Sildenafil Citrate Administration

**Published:** 2012

**Authors:** Bahman Rashidi, Jafar i Soleimani Rad, Leila Roshangar, Rafie Alizadeh Miran

**Affiliations:** 1 *Department of Anatomical Sciences and Molecular Biology, School of Medicine, Isfahan University of MedicalScience, Isfahan, Iran *; 2 *Departments of Anatomy and Histology, School of Medicine, Tabriz University of MedicalScience, Tabriz, Iran*; 3 *Drugs Applied Research Centre, Tabriz University of Medical Science, Tabriz, Iran *; 4 *Department of Anatomical Sciences, School of Medicine, Tehran University of MedicalScience, Tehran, Iran*

**Keywords:** Ovarian stimulation, Pinopode, Sildenafil citrate, Transmission electron microscopy

## Abstract

**Objective(s):**

Sildenafil citrate is a new drug and has special properties that bring about nitric oxide effects on vascular smooth muscle. The aim of this study was to assess the effects of ovarian stimulation and sildenafil citrate injection on pinopode expression in mice.

**Materials and Methods:**

Thirty adult female mice were randomly divided into three groups: control, hyperstimulated and hyperstimulated +sildenafil citrate injection. In experimental groups mice received 7.5 IU human menopausal gonadotropin (HMG) and then after 48 hr received 7.5 IU human chorionic gonadotropic (HCG) hormones. After that every two females were put with one male in one cage for mating. Hyperstimulated +sildenafil citrate group were injected with sildenafil citrate (3 mg/mouse) every 24 hr for three days, after HMG injection. The animals were sacrificed by cervical dislocation 96 hr after HMG injection, and their uterine specimens (the middle one-third) were prepared for transmission electron microscope studies.

**Result:**

Electron microscopy observations showed that in the control group there were long and short microvilli while no developed pinopodes were observed, however, in the two other groups, well developed pinopodes were expressed 4 days after HMG injection.

**Conclusion:**

The results showed that hyperstimulation of mice with sildenafil citrate may be more helpful in formation of pinopodes and implantation.

## Introduction

Implantation is one of the most interesting biological events (1) and its failure remains a major problem in infertility treatment. One of the reasons for this failure is suspected to be due to impaired uterine receptivity because of high serum estradiol concentrations induced by ovulation induction treatments (2).

One specific morphologic marker that has been proposed to be associated with the window of implantation is the appearance of pinopodes (3). Pinopodes are surface projections of the endometrial cells involved in uterine pinocytosis in mice and rats, but not in cows, humans or rabbits (4). Presence and development of pinopodes mainly depend on the ovarian hormones, especially progesterone (5). Pinopodes on the endometrial surface have been suggested to be ultrastructural markers of the implantation window (6). The surfaces of pinopodes may have some receptors for adhesion molecules, which are essential for embryo implantation. In mice, ovarian hyperstimulation is known to have a detrimental effect on the formation and disappearance of pinopodes (4). Administration of exogenous gonadotropic hormones such as human menopausal gonadotropin) )HMG ( and human chorionic gonadotropic (HCG) leads to increased secretion of estrogen and progesterone. Steroid hormones and their receptors have been suggested to be involved in the regulation of pinopode formation (7). Implantation failure remains an unsolved problem in reproductive medicine and is considered as a major cause of infertility in otherwise healthy women (8). The vasodilatory effect of sildenafil may also improve perfusion of uterus and ovaries after application to women with poor endometrial response and reduced uterine blood flow (9).

Phosphodiesterase (PDE) is a family of isoenzymes that hydrolyzes cAMP and cGMP. Specific inhibitors of PDE subtypes have been identified that can augment the effects of cyclic nucleotides on target tissues, such as human spermatozoa (10). Sildenafil citrate (Viagra) is a newly developed, type 5-specific PDE inhibitor that prevents the breakdown of cGMP and potentiates the effects of NO (Nitric Oxide) on vascular smooth muscle (10). Since its introduction in 1997, sildenafil has been used with great success in the treatment of male erectile dysfunction (11), but fewer researches have evaluated its effects in woman. The availability of sildenafil has enabled us to reap the benefits of NO on the uterus, while minimizing its side effects (10). Effects of sildenafil on perfusion of uterus and ovaries are controversial. Early reports on the benefit of sildenafil in assisted reproduction should be evaluated by placebo-controlled studies (9). In addition; there may be a role for sildenafil in the treatment of female sexual dysfunction (12). The aim of the present study was to find out whether mice uterine pinopodes are affected ultrastructurally by treatment with sildenafil citrate during immediately before implantation (13).

## Materials and Methods

***Animals***

Thirty adult (3.5 months) Syrian female and 20 adult Syrian male mice (mean weight, 25±5 g) were kept under standard laboratory conditions. The mice were acclimatized for 1 week under a 12 hr: 12 hr light: dark cycle at room temperature of 22±2 °C. Female mice were randomly divided into 3 groups.

***Group A***

*Non-stimulated control group*

A total number of 10 mice were placed in the control group and, every 2 female mice were placed with 1 male mouse in one cage, for mating.

***Group B***

*Hyperstimulated group*

The mice of this group (n=10) were hyperstimulated by intraperitoneal (IP) injection of 7.5 IU HMG (Menogon, Ferring, Pharmaceuticals, Germany) and HCG (PREGNYL, Organon, Netherlands) at an interval of 48 hr. 

***Group C***

*Hyperstimulated and sildenafil citrate administrated group*

In this group the mice (n=10) were hyperstimulated and mated in the same way as group B, then they received IP injections of sildenafil citrate (3 mg/mouse) (Rouz Darou, Iran) at 24, 48 and 72 hr interval after HMG injection.

***Tissue preparation***

Ninety six hr after HMG injection, the mice in experimental groups together with control group mice were sacrificed and their uterine were flashed for blastocyst. Uterine specimen, only from those whose uterine contained blastocyst (at least 10 in each group) was prepared (the middle one-third) transmission electron microscope (TEM) for as follows: 

Samples were washed by normal saline and afterwards were washed with phosphate buffer for 10 min (three times) and were cut into 1×1×1mm by scalpel (all samples were taken from the middle third of the uterus mice from both sides). Tissues were fixed with 2% glutaraldehyde in buffered paraformaldeide (Thuringowa, Australia) at room temperature for 30 min, and at 4 °C overnight, and then post-fixed in 1% osmium tetroxide for 2 hr at dark room. Specimens were washed with 0.1 M phosphate buffer (pH=7.4) for 10 min (three times). The tissues were dehydrated by increasing concentrations (30 to 100%) of ethanol followed by propylene oxide then were embedded in pure resin. Semithin sections (500 nm thick) were stained with 2% toluidine blue. Ultrathin sections (90-150 nm thick) (ultramicrotome, Zeiss, Germany) were stained with uranyl acetate and aqueous lead citrate. The sections were examined using Zeiss transmission electron microscope (LEO 906, Germany).

## Results

***Non-stimulated control group***

**Figure 1:**
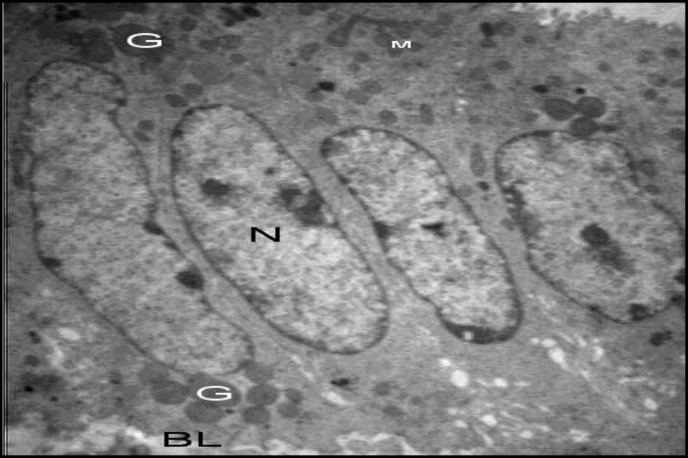
Transmission electron microscopy examination of the luminal epithelium cells 96 hr after HMG injection. N (Nucleus), G (Granule), BL (Basal lamina), M (Mitochondria). These organelles were almost similar in three groups (3592 X)

**Figure 2:**
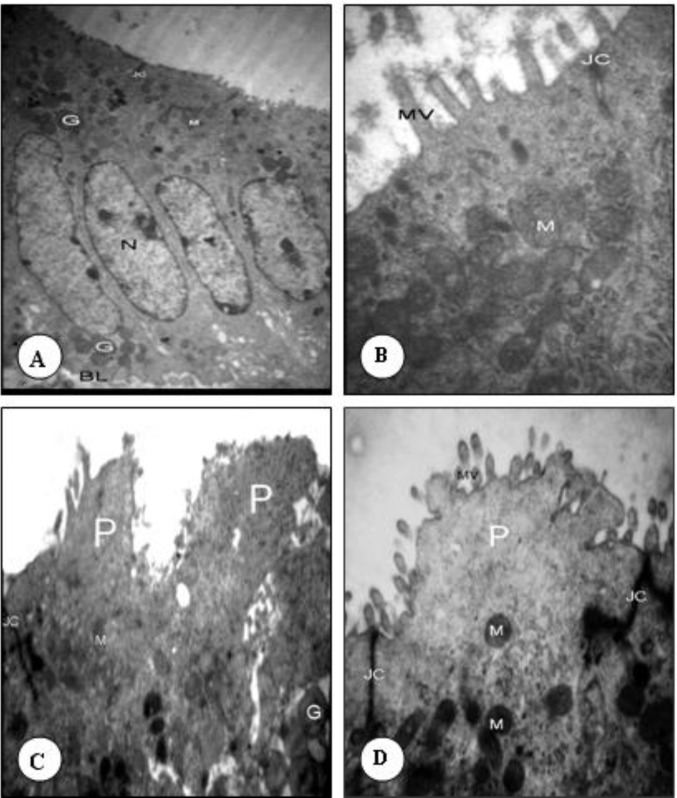
Transmission electron microscopy examination of the luminal epithelium cells 96 hr after HMG injection. A& B: non- stimulated control group, C: Hyperstimulated group, D: Hyperstimulated + sildenafil citrate injection group. MV (Microvilli), M (Mitochondria), JC (Junctional complex), P (Pinopode)

In the non- hyperstimulated mice of group A, epithelial cells (luminal) of uterine were oval with euchromatin nuclei surrounded by thin marginal heterochromatin. Their cytoplasm was rich in organelles such as rough endoplasmic reticulum, polyribosome and mitochondria. The microvilli of the uterine epithelium cells were abundant and large, 96 hr after mating (Figure 1 and Figure 2. A). No developed pinopodes in luminal cells microvillis were observed in this group (Figure 1).

***Hyperstimulated*** ***group***

In the hyperstimulated mice of group B, 96 hr after HMG injection, the microvilli decreased in number and length. Smooth and slender membrane projections form, arising from the entire cell apex (developing pinopodes). Some cells had no microvilli and were transformed to pinopodes. In this group the pinopodes were well developed and some of them had short microvilli on their surface. The nuclei of this group like group A, were oval and mainly euchromatic with a prominent nucleolus and heterochromatin dispersed peripherally; and the cytoplasmic organelles were found in the cells (Figure 2. C).

***Hyperstimulated*** ***+*** ***sildenafil citrate*** ***injected group***

In the hyperstimulated + sildenafil citrate injected groups (group C) 96 hr after HMG injection the short microvilli covered with glycocalyx were seen. In this group the apical surface of cells were similar to stimulated group and different from non-stimulated one. The euchromatic and oval nuclei with distinctive nucleoli and double membranes were situated at the mid -part of the cells. The organelles of the samples from hyperstimulated + sildenafil citrate injected animals were the same as control group (Figure 2. D).

## Discussion

The surface of endometrium undergoes cyclic changes which influences the embryo attachment and implantation. High levels of hormone produced during ovarian hyperstimulation, may disturb the structure of the endometrium (5). The relative contributions of the endometrium for successful implantation are not known, and there are no accepted criteria for the evaluation of endometrial receptivity (14). The presence and development of pinopodes is dependent on the ovarian hormones, especially progesterone (15). The premature appearance of pinopodes after hyperstimulation has been reported in some studies, but Nikas *et al* showed that the ovarian stimulation did not affect the quantity and life span of the endometrial pinopodes in human (16).

Our ultrastructural studies showed that 96 hr after mating or HMG injection, pinopodes were not visible in the control group and all the epithelial cells had microvilli on the apical surface. But hyperstimulated group had pinopodes at the same time. The pinopodes were observed for a short time, 24 to 48 hr, during implantation in mammals, (17) depending on the ovarian hormones, especially progesterone (18).

Kolb *et al* reported that although the ultrastructural features of the endometrium in the luteal phase of the ovarian hyperstimolation are better than natural phases, but may shift the window of implantation. (6). In contrast, there are some reports on a high incidence of dysfunction of endometrium under high physiological level of estrogen and progesterone. The high level of these hormones could affect the endometrial receptivity (5). Ertzeid reported that ovarian stimulation impairs implantation and fetal development in mice (14). Previous researches showed a delay in maturation of endometrium epithelium and stroma after ovarian stimulation in human and animals (19).

Mice have commonly been used as animal models in reproductive development research (20). Within the last few years, sildenafil citrate (Viagra) has been used successfully for the treatment of penile erectile dysfunction (11). Sildenafil citrate promotes smooth muscle relaxation by preventing the degradation of the second messenger cGMP by phosphodiesterase, PDE5 (12). The results showed that developed pinopodes are visible in group receiving sildenafil citrate 96 hr after injection HMG. This may either be a direct effect of the drug on the endometriom, such as inhibition of type 5-specific phosphodiesterase or potential effects of NO on vascular smooth muscle.

Using a cross-over study design, Sher and Fisch demonstrated the ability of sildenafil to modulate uterine artery blood flow and improve endometrial pattern and thickness. While improving uterine blood flow in the proliferative phase, NO may have detrimental effects on the level of the endometrium during the implantation window (10). But Barroso *et al* showed that the NO mediated release of cytokines such as tumour necrosis factor-α from activated natural killer cells have been implicated as a cause of implantation failure (21). It may be beneficial to minimize endometrial NO exposure at the time of embryo transfer and we suggest that discontinuing sildenafil citrate administrated 24 and 72 hr before the day of HCG administration, has improvs effects on pinopodes formation.

It was Barroso *et al* who, for the first time, demonstrated that higher concentrations of NO inhibit both embryo development *in vitro* and implantation *in vivo* in mice. Embryos fail to implant if either the uterine receptivity or the development of embryos is impaired, while at higher concentrations, NO is cytotoxic (21). Since Sher and Fish investigation, we have used sildenafil to improve the uterine artery blood flow and endometrial pinopodes and thus, uterine receptivity (10). 

## Conclusions

To conclude, our study on mice endometrial samples shows that after administration of HMG & HCG and sildenafil citrate, the well organized pinopodes were expressed over the surface of mouse endometrium. It seems that ovarian hyperstimulation by sildenafil citrate injection in mice could cause premature expression of pinopodes on the pre implantation time. It seems that sildenafil citrate injection may be more helpful and may increase the pregnancy rate; however, further studies are needed.
